# The Effect of Exercise in PCOS Women Who Exercise Regularly

**DOI:** 10.5812/asjsm.34874

**Published:** 2010-03

**Authors:** Afsaneh Khademi, Ashraf Alleyassin, Marzieh Aghahosseini, Leila Tabatabaeefar, Mehrnoosh Amini

**Affiliations:** 1 Sports Medicine Research Center, Tehran University of Medical Sciences, Tehran, IR Iran; 2 Department of Obstetrics and Gynecology, School of Medicine, Tehran University of Medical Sciences, Tehran, IR Iran

**Keywords:** Obesity, Woman, Polycystic ovary syndrome, Exercise, PCO

## Abstract

**Purpose:**

To determine the prevalence of polycystic ovary syndrome (PCOS) in women who exercise regularly.

**Methods:**

All women under age 45 from an industrial company who had past history of exercising more than 6 months enrolled in this cross-sectional study. Prevalence of PCOS and comparison of BMI between PCOS and non-PCOS subgroups was done. The diagnosis of PCOS was based on the revised 2003 Rotterdam ESHRE/ASRM consensus criteria and exclusion of related disorders.

**Results:**

The prevalence of PCOS in was 8.8%; 95% CI: 8.5%-9.1%. In obese subjects, mean BMI differed significantly between PCOS and non-PCOS women (29.3 ±3.3 kg/m^2^ vs. 27.8 ± 2 kg/m^2^, *P*=0.03). In lean subjects, there was no statistically significant difference in terms of BMI between PCOS and non-PCOS women (21.4 ± 1.9 kg/m^2^ vs. 21.2 ± 2 kg/m^2^, *P>*0.05).

**Conclusion:**

Obese PCOS patients show more difficulty in losing weight by exercise than lean PCOS patients. The role of hormonal alterations and PCOS per se in the responsiveness of weight loss to exercise remains to be determined.

## INTRODUCTION

Polycystic ovary syndrome (PCOS) is a complex heterogeneous endocrine disorder. It is a common disorder affecting 4-12% of women of reproductive age^[1, 2]^. PCOS was first described in the United States in 1935^[3]^. PCOS is characterized by chronic anovulation and hyperandrogenism in the absence of underlying adrenal or pituitary disease. Women with PCOS may complain about variable clinical manifestations including oligomenorrhea, hirsutism, acne, and infertility ^[4]^. Approximately 75% of these women suffer from infertility due to anovulation. Therefore, it is the most common cause of anovulatory infertility ^[5, 6]^. PCOS is also reported to be associated with obesity, insulin resistance and type II diabetes, dyslipidemia, hypertension, cardiovascular disease and endometrial carcinoma ^[7,8,9]^. Approximately 50-60% of women with the syndrome are overweight or obese compared to 30% of women in the general population^[10, 11]^.

Treatment of PCOS must focus both on normalizing short-term signs of hyperandrogenism and anovulation and on reducing metabolic complications. This can be achieved through pharmacological intervention or preferably lifestyle modification^[12]^. The most preferred and effective method of treatment of PCOS is lifestyle modification. Weight loss is an important treatment strategy.

Weight loss improves practically every parameter of PCOS. In obese, anovulatory PCOS women, weight loss restores ovulation and pregnancy rates, decreases insulin levels, diminishes acanthosis nigricans, lowers testosterone levels while raising sex hormone binding globulin (SHBG) levels, and improves psychological considerations ^[13, 14]^.

To our knowledge, there is no study to evaluate the prevalence of PCOS in women who regularly exercise. Our main objective was to estimate the frequency of PCOS in physically active women and to determine the effect of exercise on body mass index (BMI) in this group.

## METHODS AND SUBJECTS

This cross-sectional study was carried out from January 2005 to January 2006 at Tehran University of Medical Sciences, Tehran, Islamic Republic of Iran. The participants were female employees of a company who had regular physical activities.

Inclusion criteria were consisted of being less than 45 years of age and having past history of exercising more than six months. Exclusion criterion was the presence of any serious medical condition. The study group consisted of 294 women aged between 20 and 45 years who had regular physical activities.

Subjects completed an interviewer-administered questionnaire. The questionnaire contained questions on demographic data, medication usage, and personal medical history. Demographic data collected included age, education, menarche, type of exercise, duration of physical activity (expressed in hours per week), and length of physical activity (expressed in months). During the visit, participants’ heights and weights were measured, and BMI (in kg/m^2^) was calculated as weight (in kg) divided by height (in m) squared.

We used past medical history and laboratory data for cases with previous diagnosis of PCOS. A complete workup for screening of PCOS was performed in other participants. Accordingly, prevalence of PCOS was calculated.

**Diagnosis of PCOS:** The diagnosis of PCOS was made according to the revised 2003 Rotterdam ESHRE/ASRM consensus criteria and exclusion of related disorders ^[15]^. Data collected from past medical history of participants with previously established diagnosis of PCOS and current screening of other participants was the basis of making the diagnosis of PCOS. Oligoamenorrhea was defined as eight or less menses per year. Sonographic diagnosis of PCO was confirmed upon the condition of having 12 or more follicular cysts, 2-9 mm in diameter and/or increased ovarian volume (>10 ml^3^) ^[16]^. Clinical evidence of hyperandrogenism was a Ferriman–Gallwey score ≥ 8, indicating hirsutism and/or presence of moderate to severe acne^[17]^. Severe acne was characterized by presence of inflammatory nodules, in addition to comedones, papules, and pustules. Hyperandrogenemia was defined as an androgen level above normal values [a total testosterone ≥ 2.94 nmol/liter (88 ng/dl) or dehydroepi- androsterone sulfate (DHEAS) ≥ 6.64 mol/liter (2750 ng/ml)] to screen participants with one of the two criteria of oligomenorrhea or polycystic ovaries in ultrasound and without clinical signs of hyperandrogenism ^[18]^.

Other medical illnesses such as hypothyroidism, hyperprolactinaemia, congenital adrenal hyperplasia, Cushing's syndrome, androgen-secreting tumors, non-classical congenital adrenal hyperplasia were excluded ^[19]^.

**Statistical analysis:** The statistical analysis was performed using the Statistical Package for Social Sciences, version 10.0 (SPSS Inc., Chicago, IL). Data were expressed as mean ±SD (for parametric variables) and median, 10^th^, and 90^th^ percentile (for nonparametric variables), and percentile of total as appropriate. The primary outcome measure was the prevalence of PCOS in physically active women. We analyzed BMI in PCOS and non-PCOS subjects to determine the influence of exercise on BMI. The tests used were unpaired t-test, Mann-Whitney U test, and chi-square test. *P<*0.05 was considered statistically significant. Ethical approval was obtained from Ethics Committee of Tehran University of Medical Sciences. Written consent was obtained from each participant.

## RESULTS

A total number of 294 women were entered into the study. Demographic characteristics of the studied women are presented in Table 1.

**Table 1:** Demographic characteristics of subjects (PCOS and non-PCOS groups)

Characteristic	Definition	No=294	Mean (±SD) or%
**Body mass index (kg/m^2^)**			22.8 ± 3.6
**Irregular menses**		33/294	14.9%
**Score of hirsutism**			6.8 ± 3.3
**Degree of acne**	Negative	166/294	56.5%
	Mild	81/294	27.6%
	Moderate	36/294	12.2%
	severe	11/294	3.7%
**Type of exercise**	Regular walking	141/294	47.9%
	Other types of exercise	153/294	52.1%

Age range of the subjects was from 20 to 45 years with a mean (±SD) of 33.3 (± 5.6) years. Menarche ranged from 9 to 17 years old with a mean (±SD) of 12.7 (± 1.4) years. Among 44 patients with oligoamenorrhea or amenorrhea, one suffered from hypothalamic amenorrhea. The range of BMI was 16.8-34.9 kg/m^2^. The score of hirsutism ranged from 0 to 18. A total of 87 (29.6%) participants had hirsutism score ≥ eight. 16% of the participants had moderate to severe degrees of acne. One or both polycystic ovaries in ultrasound investigation were found in 48 subjects. Among this group, 21 (43.8%) patients were categorized as having PCOS.

The range of exercise time was 1 to 7 hours/week with a mean (±SD) of 3.0 (± 1.5) hours. The participants had a past history of exercising from 6 to 60 months with a mean (±SD) of 15.4 (± 11.0) months. Type of exercise was regular walking in 47.9% and other types of exercise such as swimming or aerobics in 52.1% of the participants.

PCOS was confirmed in 26 (8.8%; 95% CI: 8.5%- 9.1%) participants. Confirmation of PCOS was based on oligoamenorrhea and polycystic ovaries in four patients. In 14 patients, confirmation of PCOS was based on existence of oligoamenorrhea, clinical and/or biochemical hyperandrogenism and polycystic ovaries. Three patients with a diagnosis of PCOS had regular menses and diagnosis of PCOS was based on clinical and/or biochemical hyperandrogenism and polycystic ovaries. In 5 patients, the ovaries were in the normal range in ultrasound investigation, however they fell into the category of PCOS due to the presence of oligoamenorrhea and clinical and/or biochemical hyperandrogenism. As hirsutism was defined as Ferriman–Gallwey score ≥ eight, 57.7% (15/26) of PCOS patients were hirsute whereas hirsutism was found in 26.9% (72/268) of non-PCOS patients *(P*=0.001). The prevalence of moderate or severe acne was higher in PCOS group (30.8%) than non-PCOS group (14.6%) (*P*=0.03).

Our study population included two groups, PCOS (26 cases) and non-PCOS (268 cases). The differences of variables were analyzed between PCOS and non-PCOS subgroups (Table 2). A total of 42.3% (11/26) PCOS patients had BMI over 25 kg/m^2^, while the prevalence of high BMI was 20.5% (55/268) in non-PCOS group (*P*=0.01).

**Table 2:** Comparison of demographic characteristics between PCOS and non-PCOS subjects

Parameters	PCOS (n=26)	Non-PCOS (n=268)	*P Value*
**Mean age (y)**	31 (22.7-39)	33 (28-42)	0.007*
**Mean menarche (y)**	12.7±0.9	12.6±1.4	NS‡
**BMI (kg/m^2^)**	23.8 (19-33.4)	22.02 (18.5-27.4)	0.03*
**Current use of oral contraceptive pills**	7.7%	9.1%	NS#
**Duration of exercise (hours)**	31.5 (8-108)	26 (12-90)	NS*
**Regular walking**	49.1%	44.5%	NS#

* Mann-Whitney U test

‡ Unpaired t-test

# Chi-square test

NS= Non-significant

We divided participants into two subgroups based on BMI. Among obese subjects (BMI > 25 kg/m^2^), the prevalence of PCOS was 16.6% (11/66). Using unpaired t-test, in obese subjects mean BMI differed significantly between PCOS and non-PCOS patients (29.3±3.3 kg/m^2^ versus 27.8±2 kg/m^2^; *P* = 0.03). The prevalence of PCOS was 7% (15/213) among lean subjects (BMI ≤ 25 kg/m^2^). In lean subjects there was no statistically significant difference in terms of BMI between PCOS and non-PCOS patients (21.4±1.9 kg/m^2^ vs. 21.2±2 kg/m^2^; *P*>0.05).

## DISCUSSION

The main outcome of this study was to estimate the frequency of PCOS in a selected group of females. We reported the prevalence of PCOS in adult women who exercise regularly, using the new Rotterdam Consensus. In our study, PCOS was detected in 8.8% of participants.

It is emphasized that the most preferred and most effective method of treatment for PCOS is lifestyle modification ^[20]^. Nevertheless, how much lifestyle modifications are practical in PCOS patients?

Randeva et al showed that exercise, such as regular walking, reduces waist-to-hip ratio, an indicator of diabetes and other morbidities, and homocysteine levels, an indicator of cardiovascular risk, in overweight PCOS women. However only half (12/21) of their subjects completed the exercise program^[21]^.

In a study, researches offered 33 obese PCOS patients a 1200 kcal/day diet. Patients were simply advised to do some swimming or aerobics at least once or twice a week though no information was reported as to the actual duration and/or intensity achieved. They emphasized that not directly ascertain if the prescribed diet was followed. A total of 24% of their patients failed to lose weight. Only 33% achieved 10% decrease of body weight ^[22]^. It seems that losing weight by lifestyle modifications is difficult in a special subgroup of PCOS patients.

Although weight loss improves practically every parameter of PCOS, Wright et al. concluded that differences in dietary intake and physical activity alone are not sufficient to explain differences in weight between women with and without PCOS ^[23]^. Our study confirms the findings by Wright et al. about lifestyle. We showed that although the mean time of exercise did not differ significantly between PCOS and non-PCOS subgroups, frequency of obesity in women with PCOS was higher than non-PCOS subgroup of the same sample. This difference could be contributed to interaction between metabolic disturbances and lifestyles such as dietary intake and exercise.

Previous researchers have speculated that women with PCOS are obese due to a tendency toward overeating, particularly sweet or starchy foods ^[24]^. Faloria et al. evaluated the effect, if any, of obesity on metabolic features, body composition and fat distribution of patients with PCOS ^[25]^. They showed that none of the lean subjects suffered from metabolic syndrome as opposed to 37% of overweight-obese patients and 33.3% of overweight-obese control subjects. It seems that metabolic mechanisms act differently between lean and obese subgroups. Therefore we divided our sample to lean and obese subgroups.

We showed that mean BMI difference between PCOS and non-PCOS obese subjects was statistically significant, although the difference was not statistically significant between lean PCOS and non-PCOS subjects. It seems that obese PCOS patients had some negative factors which acted against long term maintenance of weight loss. Wright et al. results confirm our findings. After dividing the study population to lean and obese subgroups, they concluded that energy intake of normal-weight women with PCOS was significantly lower than that of normal-weight women without PCOS. Conversely, the energy intake of obese women with PCOS was greater than that of obese women without the syndrome, although the difference was not statistically significant. They emphasized that women with PCOS should restrict significantly energy intake in order to maintain a normal weight ^[23]^. In the same way, the effect of exercise in long term could be less efficient in obese patients.

Long-term maintenance of weight loss among obese population is less likely ^[26]^. This issue is exaggerated in obese PCOS subjects due to impressive correlation between metabolic characteristics, lifestyle such as physical activity and dietary intake, and obesity.

Although lifestyle modifying measures, such as diet control and exercise, could play an important role in treatment of PCOS, adding more special programs to overcome non-compliance and to lower dropout rates of trials for weight loss is necessary^[27]^. Further research is necessary to determine the relative contributions of lifestyle including exercise and dietary intake, and PCOS. Designing cohort studies to determine which factors influence weight loss and/or long term maintenance of weight loss in PCOS patients will clarify the degree of exercise effectiveness.

## CONCLUSION

In this study, mean BMI differed significantly between PCOS and non-PCOS women. Obese PCOS patients show more difficulty in losing weight by exercise than lean PCOS patients. The role of hormonal alterations and PCOS per se in the responsiveness of weight loss to exercise remains to be determined.
